# Experimental datasets on engineering properties of expansive soil treated with common salt

**DOI:** 10.1016/j.dib.2018.04.038

**Published:** 2018-04-14

**Authors:** Taiwo O. Durotoye, Joseph O. Akinmusuru, Kunle E. Ogundipe

**Affiliations:** a Department of Civil Engineering, Covenant University, Ota, Nigeria; b Department of Building Technology, Covenant University, Ota, Nigeria

**Keywords:** Common salt, Expansive soil, Experimental procedure, Strength parameters, Swelling parameters

## Abstract

Construction of highway pavements or high rise structures over the expansive soils are always problematic due to failures of volume change or swelling characteristic experienced in the water permeability of the soil. The data in this article represented summary of (Durotoye et al., 2016; Durotoye, 2016) [1], [2]. The data explored different percentages of sodium chloride as additive in stabilizing the engineering properties of expansive soil compared with other available stabilizer previously worked on. Experimental procedures carried out on expansive soil include: (Liquid limit, Plastic limit, Plasticity index, Shrinkage limit, Specific gravity Free swell index and Optimum water content) to determine the swelling parameters and (maximum dry density, California bearing ratio and unconfined compressive strength) to determine the strength parameters. The results of the experiment were presented in pie charts.

**Specification table**

**Table:** 

Subject area	Building Construction, Geo-Technical Engineering
More specific subject area	Highway Engineering, Foundation Engineering
Type of data	Figure, text file
How data was acquired:	The data was gotten from Laboratory and experimental procedures and simple statistical methods was used for the analyses
Data format:	Raw data obtained from experimental procedures were calculated and plotted in figures
Experimental factors:	Various test on swelling properties and strength parameters of expansive soil were carried out.
Experimental features:	Engineering properties of expansive soil and laboratory tests.
Data source location:	Oniyale, Ifo, Ewekoro Local Government Area, Ogun State, Nigeria.
Data accessibility:	The article can be assessed on public repository http://eprints.covenantuniversity.edu.ng/
Related research article:	Durotoye TO, Akinmusuru JO, Ogbiye AS, Bamigboye GO. Effect of Common Salt on the Engineering Properties of Expansive Soil”. International Journal of Engineering and Technology. 2016, 6, 7

**Value of the data**

• The data provided detailed experimental procedures on how common salt could be used to stabilize expansive soil thereby reducing its swelling properties.
• The data provided an insight into cost effective method of stabilizing expansive soil.
• The data could be useful in research that involves studying of expansive soil for the construction of highway pavement and high rise building.
• The data experimental procedure is detailed and it can be adopted or modified for further study or to compare the results of this data.

## 1 Data

Several studies has been done on the available additives to stabilized expensive soil due to its change in moisture contents and problematic in nature that causes soil to either shrink or swell under loading [1], [2], [3], [4], [5], [6], [7], [8], [9], [10], [11], [12], [13], [14], [15], [16], [17], [18], [19], [20]. However, studies conducted by [7], [8], [9], [10] established the addition of different percentages of sodium chloride as additive for stabilizing expansive soils.

**Fig. 1:**
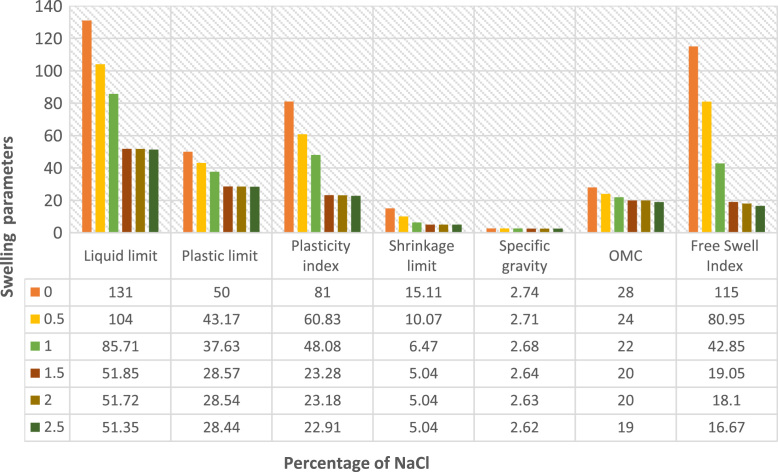
Behaviour of swelling engineering parameters treated with common salt.

**Fig. 2:**
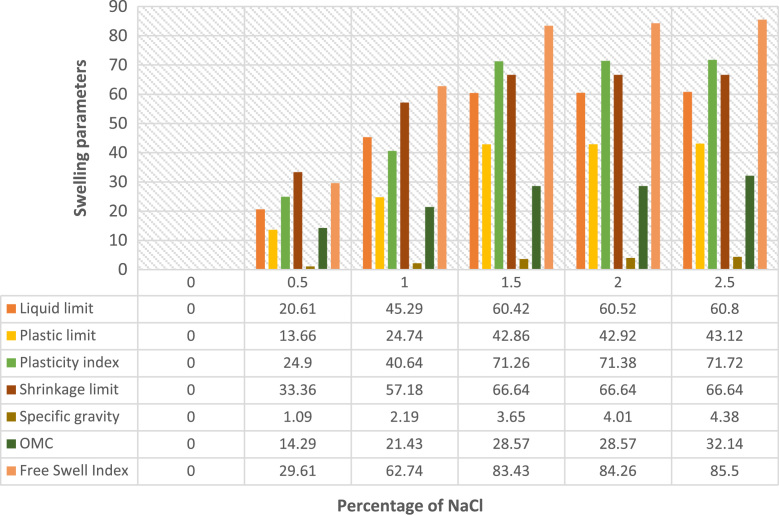
Reduction of swelling parameters with addition of common salt.

**Fig. 3:**
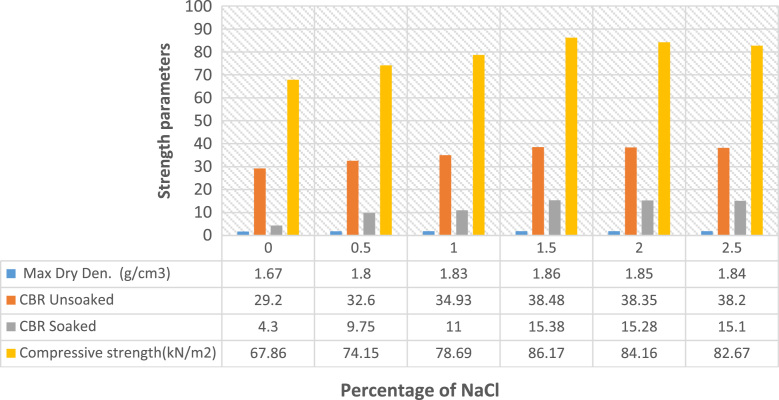
Behaviour of strength parameters treated with sodium chloride (NaCl).

**Fig. 4:**
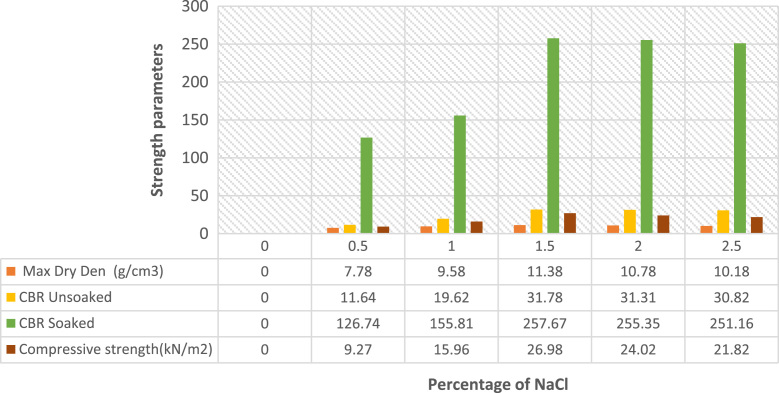
Percentage increase in strength parameters treated with sodium chloride (NaCl).

The data presented in Fig. 1, Fig. 2, Fig. 3, Fig. 4 were gotten from the analyses of swelling and strength parameters of expansive soil with the addition of common salt in order to make it more suitable for construction activities. The behaviour of sodium chloride on swelling parameters of expansive soil are shown in Fig. 1 with different percentages addition of sodium chloride. The reduction in Liquid limit, Plastic limit, Plasticity index, Shrinkage limit, Specific gravity Free swell index and Optimum water content are illustrated in Fig. 2. Data of strength parameters are shown in Fig. 3 and the relative increase in the maximum dry density, California bearing ratio unsoaked, California bearing ratio soaked and unconfined compressive strength are shown in Fig. 4.

## 2 Experimental design, materials and methods

The sample of expansive soil used for this data was gotten from highway pavement (subgrade) works at Oniyale, Ifo, Ewekoro Local Government Area, Ogun State, Nigeria. The experimental procedure were carried out in three stages. Firstly, sample gotten from the study area was air dried because the sample was gotten during the raining season. Secondly, the sample was sieved through 4.75 mm sieve to dispose rock division and the sieved soil was put into water/air proof compartments for stability. Thirdly, sample kept for stability was blended with common salt and distilled water at different percentages ranging between 0%, 0.5%, 1.0%, 1.5%, 2.0%, and 2.5%. The test conducted were classified into two Swelling parameters: (Liquid limit, Plastic limit, Plasticity index, Shrinkage limit, Specific gravity Free swell index and Optimum) and Strength parameters: (maximum dry density, California bearing ratio unsoaked, California bearing ratio soaked and unconfined compressive strength). However, various experimental procedures conducted on engineering properties of expansive soil was in accordance with British Standard codes [14].

The variety of swelling restrain with sodium chloride content on the expansive soil are shown in Fig. 1, Fig. 2**,** shrinkage restrict drastically reduced the swelling parameters with an increase in percentage of sodium chloride content [8], [15]. Fig. 3, Fig. 4 show improvements in strength parameters of the treated soil with the additions of sodium chloride [7], [8]. Thereby, the Maximum dry density, California bearing proportion and the unconfined compressive quality were increased and made the sample more stabilized. The data presented proof to be cost effective when compared with previous studies on expansive soil [11], [12], [13], [15], [16], [17], [18], [19], [20]. It also exemplified safety knowledge and practices needed to be considered in the process [21], [22].
